# Use of Oral Cholera Vaccine in Complex Emergencies: What Next? Summary Report of an Expert Meeting and Recommendations of WHO

**Published:** 2007-06

**Authors:** Claire-Lise Chaignat, Victoria Monti

**Affiliations:** Global Task Force on Cholera Control, World Health Organization, Avenue Appia 20, CH-1211 Geneva 27, Switzerland

**Keywords:** Cholera, Cholera vaccines, Evaluation studies, Complex emergencies

## Abstract

Two meetings of the World Health Organization (WHO)—in 1999 and 2002—had examined the potential use of oral cholera vaccines (OCVs) as an additional public-health tool for the control of cholera. In the light of the work accomplished since 2002, WHO convened a third meeting to reexamine with a group of experts the role that OCVs might play in preventing potential outbreaks of cholera in crisis situations and to discuss the use of OCVs in endemic settings. The aim of the meeting was to agree a framework for the recommendations of WHO on these subjects and to consider the pertinence of further demonstration projects in endemic settings. The meeting addressed key issues, including currently-available vaccines, surveillance, and cholera-control measures in complex emergencies, and past experiences of using OCVs. More than 40 participants took part in the discussions, representing cholera-prone countries, humanitarian organizations, scientific institutions, United Nations agencies, and WHO. The experts agreed that when considering the use of OCVs in emergencies, a multidisciplinary approach is essential and that the prevention and control of cholera should be envisaged within the larger context of public-health priorities in times of crisis. As for the use of OCVs in endemic settings, all participants acknowledged that further data need to be collected before a clear definition of endemicity and potential vaccination strategies can be established. Results of further studies on the vaccines per se are also awaited. Recommendations relating to the use of OCVs (a) in complex emergencies and (b) in endemic settings were elaborated, and a decision-making tool for assessing the pertinence of use of OCVs in emergency settings was drafted. The document was finalized by an ad-hoc working group convened in Geneva on 1 March 2006 and is now available for field-testing. After testing, that should be carried out with the involvement of WHO and feedback from field partners, the decision-making tool will be adapted and disseminated.

## INTRODUCTION

Although well-known since the nineteenth century, cholera remains the most feared and stigmatized diarrhoeal disease. As a waterborne disease, it mainly affects the poorest and the most vulnerable populations who live without access to safe water and proper sanitation. The burden it imposes on healthcare systems and on its victims is enormous. Furthermore, countries, fearful of possible commercial sanctions that would prevent the export of food products, are often reluctant to report cases and seek support. Heavy death tolls are regularly reported and, in disaster situations, the possibility of cholera frequently triggers panic—even when the risk of outbreak appears extremely limited.

Implementation of the prevention and control measures usually recommended, including improvement of water and sanitation, remains a challenge in both urban slums and crisis situations. To date, there has been no concrete global improvement despite efforts made at the country level; the incidence of disease has even increased in recent years. Predicting potential outbreaks remains difficult and is often complicated by the lack of data on trends and patterns of the disease over time.

It is clear that additional public-health tools, such as vaccines, can play a critical role in the control of cholera. The pre-emptive use of oral cholera vaccines (OCVs) in emergency situations was recommended by the World Health Organization (WHO) in 1999, and this general recommendation remains valid (1,2). However, vaccines must be used in appropriate circumstances, where they can provide a definite benefit and will not jeopardize the response to other health priorities. Identifying the population at risk of epidemic cholera is, therefore, a key element in considering the use of OCVs, as is the cost-effectiveness of such an intervention. Several mass-vaccination campaigns have already been carried out in crisis situations, and a group of experts, convened in a WHO meeting, used the evidence provided by these interventions as the basis for developing assessment tools and recommendations for the use of OCVs in mass-vaccination campaigns and to identify the possible constraints and limitations. This meeting, held in Cairo, Egypt, on 14-16 December 2005, intended to establish a framework for recommendations on the use of OCVs in complex emergencies and natural disasters and in endemic settings (3). More than 40 participants were present, representing cholera-prone countries that had already used or expressed interest in using OCVs, humanitarian organizations, scientific institutions, United Nations agencies, and WHO. A vaccine manufacturer was granted an observer status, but did not attend sessions aimed at developing recommendations on the use of OCVs.

### Available vaccines and new developments

Because of its low-protective efficacy and the frequent occurrence of severe adverse reactions, the early parenteral cholera vaccine was never recommended for use (4). To date, two oral vaccines have been licensed internationally. One consists of killed whole-cell Vibrio cholerae O1 with purified recombinant B-subunit of cholera toxin (WC/rBS). It is administered in two doses, with an interval of 10-14 days between the doses. A large volume (75-150 mL) of liquid is needed for administration, meaning that the vaccine cannot be given to children aged less than two years. Protection starts 10 days after the ingestion of the second dose and has been shown to reach 85-90% after six months in all age-groups, declining to 62% at one year among adults (5). This vaccine, currently produced in Sweden, has been granted WHO prequalification.

The second licensed vaccine consists of an attenuated, live, and genetically-modified V. cholerae O1 strain (CVD 103-HgR) (6). It is administered in a single dose to individuals aged two years and over; protection starts eight days after ingestion (7). Although a 95% seroconversion and protection was observed during a challenge study, a large field trial undertaken in Indonesia, in circumstances that complicated interpretation, failed to demonstrate convincing protection (8). The manufacturer stopped production in 2004, and the vaccine, although licensed, is currently unavailable.

Technology transfer to Viet Nam has generated a variant of the killed whole-cell vaccine containing no recombinant B-subunit, i.e. WC vaccine. This vaccine, currently produced and used only in Viet Nam, is given in two doses at an interval of 10-14 days, without the need for a buffer solution. The protective efficacy of a first-generation monovalent (anti-O1) Vietnamese cholera vaccine was shown to be 66% (68% in children) 8-10 months after vaccination (9). Killed O139 whole cells were added to the Vietnamese vaccine following the emergence of the new form of epidemic cholera caused by this serogroup. A study found the bivalent vaccine to be safe and immunogenic in adults and children aged one year and older (10,11). Technology transfer to India, that could lead to a WHO prequalification, is underway.

A number of other live oral vaccines are under development in the USA (12) and in Cuba (13). In addition, research is currently being conducted on parenteral conjugate vaccines and on ways to improve vaccine formulation to ease the numerous logistics constraints, particularly acute in emergencies, linked to the mode of administration of the vaccine presently available. Indeed, the limitations of the WC/rBC vaccine in emergency settings, where logistic and practical constraints abound, are numerous, but its use in a routine context is much more easily managed (14). Since efficacy requirements may be lower in an emergency context, vaccines specifically designed for emergency public-health applications might be considered (15,16).

### Potential effect of herd protection

In researching the public-health impact of cholera immunization, the concepts of herd protection and herd amplification, which arose from recent environmental studies, are important issues that merit examination. When dealing with a killed vaccine, the term herd protection is preferred to herd immunity as unvaccinated persons do not develop antibodies. If these concepts prove to be sound, herd protection may have a major role in increasing the impact of vaccination and reducing the cost and burden of cholera—factors that are essential elements in any consideration of the future use of cholera vaccines.

A new analysis of the 1985 cholera vaccine trial in Bangladesh established that there was an additional indirect protective effect among both vaccinated and non-vaccinated individuals when a high proportion of the population was vaccinated and a possible reduction of the incidence of cholera in all age-groups (17). The public-health impact of killed OCV may, thus, have been underestimated in the past, as only the conventional protection efficacy of the vaccine was measured and not the potential effect of herd protection. Further studies are, therefore, needed to precisely evaluate the effect of herd protection, especially as a number of circumstances can have induced a bias (density of population and dwellings, environmental factors, health-education programmes, and microbiological aspects of the disease) (18).

The design of future vaccine-evaluation and efficacy studies will need to consider the role of herd protection. The hypothetical existence of significant herd protection will have implications for the choice of target populations for cholera vaccination. It is likely that access to the vaccine might be enhanced for groups who do not usually have access to or seek treatment. It remains to be determined how these elements will influence the development of strategies that focus on reaching a particular threshold level of vaccination to achieve an acceptable level of protection in a community.

### Surveillance in complex emergencies

Several definitions describe the blurred concept of complex emergencies. In this meeting, a pragmatic public-health perspective was adopted, aiming principally at highlighting the health priorities and challenges. All participants agreed to define complex emergencies in the following terms:

- a large part of the population is affected, leading to potential massive movements of populations;
- coping capacities of the local and national authorities are overwhelmed by the magnitude of man-made or natural disasters; and
- numerous national and international actors may participate in the relief efforts.

The first consequence of a complex emergency is the upheaval of usual life and the emergence of ‘new’ vulnerable population groups. Lack of access to basic services and healthcare, lack of food, and displacement have an impact on the health status of the population. Restoring an acceptable health status presents a number of varied challenges: prioritization of health issues, coordination of the numerous actors involved, and timeliness when urgent action is required. The decision-making and preparatory phase is often extremely short, and access to vulnerable populations is frequently limited by specific geographical difficulties, further natural disasters, a volatile security environment, or mass population movements. People living in overcrowded camps with poor environmental status are exposed to a higher risk of transmission of cholera if V. cholerae is endemic in the area or has been introduced. Moreover, uncontrolled rumours and panic are often rife: in every catastrophe, false beliefs regarding plagues and epidemics transmitted by dead bodies tend to be widespread. In such contexts, cholera remains, rightly or wrongly, the disease most feared by the population and by the authorities.

In all cases, the occurrence, spread, and extent of an outbreak of cholera are extremely difficult to predict. They depend on a multiplicity of aspects, including local endemicity, living conditions, forced or voluntary movements of population, environmental and cultural factors, and the effectiveness of any control measures put in place. In some endemic situations, where outbreaks tend to occur at regular intervals, seasonal recrudescence can be anticipated, provided that enough epidemiological data are known. The establishment of an epidemiological surveillance system that will provide baseline data and trends is, thus, a key element in directing the potential use of OCVs. However, if the early warning system is a challenge in many countries, surveillance and gathering of data in a complex emergency are even more problematic. One of the main difficulties is to establish a system that is both reactive and sustainable; this is particularly tricky when resources are scarce and security cannot be ensured. For cholera, the introduction of a rapid, easy-to-use, and affordable diagnostic test, currently under development, will be critical. Nevertheless, the task is complicated by the stigma attached to the disease and the reluctance of many to report cases for fear of travel and trade sanctions, a fact that impacts negatively on surveillance.

An early warning system using standard case definition is essential to trigger the alert promptly, an element particularly critical in high-risk situations, such as in refugee camps and urban slums, and among displaced populations. The definition of outbreak should take into account essential background information, including the occurrence of previous cases or outbreaks and endemicity.

The following definitions used by Médecins sans Frontières (MSF) are a good example:

- in endemic areas: doubling of cases over three consecutive weeks or increase in cases compared to the previous year;
- in non-endemic areas: an increasing number of confirmed cases;
- an increasing number of adults dying of watery diarrhoea.

### Water and sanitation in complex emergencies

Other elements closely relating to the containment of outbreaks of cholera are water supplies and sanitation status of populations at risk. The example of Darfur, Sudan, offers valuable indications of the cost, impact, and challenges of water and sanitation projects in complex emergencies and of the role of such projects in preventing outbreaks of cholera. At the beginning of the humanitarian intervention in May 2004, only 20% of the internally-displaced persons (IDPs) living in areas reachable by the United Nations agencies had access to adequate water, and only about 5% to proper sanitation; by September 2005, 16 months later, these figures had risen to 52% and 76% respectively. These numbers are not calculated according to the Sphere standards and are provided for information only; they cannot be considered an accurate indicator of water and sanitation supplies to a population and do not describe the actual conditions faced by the people. Clearly, despite the enormous efforts provided by all humanitarian bodies active in the field for more than a year, a significant number of people still lacked access to minimum water supply and sanitation facilities. This situation serves also to illustrate the obstacles faced by humanitarian workers—lack of human and financial resources, logistic constraints, limited access to beneficiaries, and poor planning and coordination—that prevent sustained implementation and maintenance.

The exact cost of improved water and sanitation is difficult to establish; a comparison of the costs of different interventions is, therefore, needed. The cost–benefit of improved water and sanitation, from both health and socioeconomic perspectives, is seen mainly in the reduction of waterborne diseases—cholera and others—which lowers health-related costs and reduces morbidity.

### Usually recommended cholera-controlmeasures

Once an outbreak is detected, the usual intervention strategy aims at reducing mortality—ideally below 1%—by ensuring access to treatment and controlling the spread of disease. To achieve this, all partners involved should be properly coordinated and those in charge of water and sanitation must be included in the response strategy. The main tools used for the treatment of cholera are:

- proper and timely rehydration in cholera-treatment centres and oral rehydration corners;
- specific training for proper case management, including avoidance of nosocomial infections;
- sufficient prepositioned medical supplies for case management, e.g. diarrhoeal disease kits;
- improved access to water, effective sanitation, proper waste management, and vector control;
- enhanced hygiene and food safety practices; and
- improved communication and public information.

Among these measures, the provision of safe water and sanitation in emergencies is a formidable challenge but remains the critical factor in reducing the impact of outbreaks of cholera. Recommended control methods, including standardized case management, have proved to be effective in reducing the case-fatality rate. A comprehensive multidisciplinary approach should, therefore, be adopted for dealing with a potential outbreak of cholera, and the use of OCVs should be integrated when it can make a difference. Cost-effectiveness of such interventions needs clarification. Although the exact cost of different interventions is difficult to establish, they still need to be investigated and compared.

### Use of OCVs in crisis situations: recent examples

The value and potential impact of OCVs in different settings were debated on the basis of evidence accumulated since 2002. The examples of two mass-vaccination campaigns—carried out with WHO support in 2004 in Darfur and in 2005 in Aceh—were examined and compared. Both the campaigns took place during complex emergencies, but the nature of the emergencies and of the target populations, the simultaneous implementation of programmes to address other public-health priorities, the location of the campaigns, and the partners involved were widely different.

In Darfur, 87% of 53,537 people targeted—internally-displaced people accommodated in two camps where water supplies and sanitation were poor—received two doses of the WC/rBS. The campaign was completed in about six weeks, and the direct costs of the campaign reached US$ 336,527, or US$ 7 per fully-immunized person. In Aceh, 69.3% of 78,870 people initially targeted—people displaced by the tsunami and scattered around large areas—had received two doses of the same vaccine. The campaign was completed in more than six months, and the direct costs of the campaign reached US$ 958,649, or US$ 18 per fully-immunized person (19).

The evidence from Darfur indicates that a small-scale mass-vaccination campaign with OCVs is feasible provided that there is a strong political commitment, easy access to the target population, that is accommodated in closed IDP camps, widespread community mobilization, and involvement of all partners. The Aceh campaign, however, points clearly to the limitations of using a two-dose vaccine in the context of a natural disaster. Enormous logistics and operational constraints greatly delayed the implementation of the vaccination—it took more than six months to complete it—and increased the costs disproportionately. Furthermore, insufficient cold chain and the short shelf-life of vaccines led to an overall vaccine wastage of 11.7%. The feasibility of large-scale interventions is questionable: future campaigns will require solutions to the many difficulties encountered, and a suitable methodology is needed to guide the decision-making process of governments wishing to consider the use of OCVs. The main lessons learned from Aceh and Darfur can be summarized as follows:

- An OCV campaign is feasible in natural and man-made disasters, provided that political commitment and good social mobilization can be achieved, good logistics can be ensured, and sufficient funds are available. The target population should be well-defined, localized in a small area, and stable.
- A mass OCV-vaccination campaign serves to highlight important deficiencies in water and sanitation coverage and to build the commitment of stakeholders and implementing agencies.
- The use of OCV is only one part of a set of comprehensive public-health preventive interventions.

### Logistics and planning challenges for use of OCVs in crisis situations

These two recent examples show that the use of the two-dose OCV in emergency settings can be seriously challenged by various shortfalls and the onerous logistics involved. Several characteristics of the vaccine are less than ideal for emergency settings, including its shelf-life, required storage conditions (cold-chain, at between +2 °C and +8 °C), and volume (25 times greater than measles vaccine); moreover, its mode of administration demands the availability of significant volumes of clean water and requires the target population to be reached twice within a short time (10-14 days).

Although logistic constraints can often be overcome, they usually lead to delays in implementation and significant increases in cost. In each situation, the cost-benefit must be thoroughly assessed and the whole campaign planned in detail. Experience in planning and implementing mass OCV-vaccination campaigns in various settings since 1999 has helped identify the following 12 principal challenges:

- During natural disasters or other complex emergencies, basic infrastructures are damaged and disrupted, the population is vulnerable and subject to continual threats, and healthcare personnel are scarce.
- Access to target populations is often limited by geographical factors, destruction of roads, climatic conditions, potential aftershocks, and a volatile security situation.
- To deal with perceived but unconfirmed risks that may not be based on solid evidence, a risk assessment should be carried out: available epidemiological data, living conditions faced by the population, climatic conditions, environmental management, and cultural behaviours are the key elements to be examined.
- The target population may be difficult to identify with precision when there are continual population movements.
- Thorough planning and preparation are crucial: coordination with partners is important, as are the assignment of responsibilities and good logistic arrangements. Functioning communications, training of field staff, adequate health education, and social mobilization programmes are other elements to be taken into account.
- During the implementation phase, monitoring of the operations, ensuring timely delivery of supplies, and maintaining communication with community leaders are crucial.
- Logistics must be thoroughly planned and closely monitored throughout the campaign, with the principal focus on transport and storage of supplies, transport of field teams, cold-chain facilities, management of wastes, and reliable telecommunications.
- An efficient surveillance system is vital for the early detection of any cholera cases that occur after the vaccination and for the implementation of specific control measures.
- Sustained improvement in environmental management, access to safe water and proper sanitation, and adequate hygiene and food safety are essential components of a comprehensive control strategy for cholera.
- Health education constitutes a long-term effort and needs to address the vaccine itself, the vaccination campaign, and food hygiene, and water and environmental safety. Involvement of the community is critical to ensure effective social mobilization for the campaign and to avoid culturally-inappropriate activities.
- Problems with vaccine availability, affordability, and packaging (if not adequately designed) can prevent smooth implementation. Before the campaign begins, ad-hoc solutions must be found.
- The reality of a vaccination campaign inevitably differs from what was originally planned and expected. A detailed timeline helps anti-cipate potential hindrances and plan alternative solutions.

Clearly, a mass-vaccination campaign cannot be improvised at the last moment—it needs careful advance preparation. If time constraints do not allow for proper planning, for instance if an outbreak is about to start or has already started, the use of OCVs may not be appropriate. Experience shows that, once an outbreak of cholera has begun, a reactive vaccination campaign with a two-dose vaccine is almost impossible.

In addition, the use of OCVs needs to be positioned within the larger context of other public-health priorities. It should be additional to health education and improvements in water and sanitation, not the sole intervention, and should never be seen as a substitute for preparedness for outbreaks of cholera—pre-positioning of supplies for case management, health education, and improvements in water supply and sanitation. In settings where a population is inaccessible for extended periods (for example, in detention facilities) or when the water and sanitation status cannot be rapidly improved, the use of OCVs may be a definite benefit. The use of two-dose OCV is easier in closed settings (refugee and IDP camps, detention facilities, etc.), where population movements are limited and can be better controlled than in open settings, such as the spontaneous IPD settlements found in Aceh. The feasibility of scaling up interventions remains to be proved, and the cost-benefit should be further analyzed. For the time being, the two-dose vaccine and the logistics associated with its use remain very expensive.

### Use of OCVs in endemic settings

A demonstration project, carried out in Beira, Mozambique, showed that a mass-vaccination campaign using OCVs was feasible (20,21), acceptable, and effective (22) for at least six months. Around 57% of the target population—inhabitants of Esturro neighbourhood—received two doses of the WC/rBS, and a case-control study conducted in 2004, involving 43 patients with cholera, demonstrated a protective efficacy of 78%. Nevertheless, the study left a number of important questions unanswered, including duration of the protection, existence of herd protection, protection within the HIV-positive population, and cost-effectiveness.

Globally, studies carried out in different countries suggest that the proper definitions of cholera cases and of endemicity need to be further defined. Differences in methodologies and in attitudes of national authorities towards cholera can result in different approaches to the disease—including the prevention measures to be adopted and the potential use of OCVs. It is, therefore, important to find a definition of cholera endemicity that can be widely adopted. A threshold of one case per 1,000 people has been proposed, but has yet to be universally accepted. Epidemiological data still need to be collected: lack of these data is an obstacle for advocating the use of OCVs. On the other hand, increasing treatment costs and rising antimicrobial resistance make development of a vaccination strategy for endemic settings highly desirable, provided that the vaccine can be formulated for administration to children aged less than two years, can protect against both V. cholerae O1 and O139 serotypes, and is cost-effective.

Indeed, the cost per death averted and per hospitalization averted declines with the increasing incidence of cholera: even a very inexpensive vaccine becomes cost-effective only when the incidence exceeds 1/1,000. By comparison, the same model estimates that case management, if provided through routine hospital or treatment centre care, costs about US$ 350 per death averted. Even moderately-inexpensive vaccines, therefore, quickly become too expensive. For example, a vaccine requiring two doses at US$ 3 per dose will cost more than US$ 3,000 per death averted, even where the incidence is high. By contrast, a vaccine priced at US$ 0.40 will cost less than US$ 400 per death averted, which compares favourably with case management, especially as hospital and treatment costs will decrease. In addition, models have been developed to determine the key variables, the most important of which appear to be the incidence of cholera and the cost of the vaccine, including delivery cost (23). The efficacy of vaccines seems of less importance. Vaccines should, thus, be inexpensive and easy to administer and should be provided to inhabitants of high-risk areas. Furthermore, a vaccine marketed over-the-counter may be economic for health ministries, since it would shift the vaccine costs to the consumer rather than to the government. Finally, the adoption of vaccination strategies will not replace treatment facilities.

Countries interested in using OCVs in endemic settings will need to design vaccination strategies that will achieve the best possible coverage. Different strategies can be envisaged, but all should be based on mapping of risk and should take account of high-risk groups (particular age-groups and vulnerable populations living in specific geographical areas) and feasibility. The sustainability of vaccination strategies is the paramount consideration: mass-vaccination campaigns that are not sustainable may be useless and possibly counter-productive.

Experts present in the meeting recognized that a number of issues still need to be studied, including the efficacy of OCVs in populations with a high proportion of HIV-positive individuals, a definition of endemicity, and the cost-effectiveness of the vaccine. Work should also be done on vaccination strategies. Although the use of OCVs in endemic settings can be supported in principle, detailed recommendations remain to be worked out. The group recommended the synergistic use of control measures other than vaccine, namely improvement of water supply and sanitation and health education. Demonstration projects should yield additional useful data.

### Pertinence of a stockpile of cholera vaccines

The example of the International Coordinating Group on Meningitis (ICGM) for supply of antimeningococcal vaccine was taken to assess the pertinence of creating a stockpile of cholera vaccines. In view of the numerous difficulties and high financial costs involved, the advantages and disadvantages of creating a stockpile should be examined in detail, and an adequate stock rotation should be ensured.

The only OCV currently available on the international market is manufactured by the Swedish company—SBL Vaccines—under the commercial name Dukoral^¯^. To date, the vaccine is not widely used, although licensed in 45 countries. Production costs remain high and are not covered by the price of the vaccine—up to €5 (US$ 6.10) a dose. Maintained in a cold-chain, Dukoral^¯^ has a shelf-life of three years; according to the manufacturer, it can be kept at 25 °C for three months and at 37 °C for one month, but these storage conditions were not recognized in the prequalification process.

The WHO recommendations of 1999 proposed the establishment of a stockpile of two million doses of cholera vaccine for use in endemic and emergency settings. However, because of the lack of precise guidelines for the use of OCV, the high costs involved, and the limitations that became apparent during mass-vaccination projects carried out in the 2000-2005 period, the stockpile was never implemented. Moreover, the only current OCV manufacturer has clearly stated that, without firm orders, its limited production capacities will not be expanded. Thus, until recommendations and guidelines are issued and promoted, the issue of a stockpile is not relevant. The subject will be raised with partners and donors after field validation of the recommendations—and, in particular, of the decision-making tool—when countries concerned express their willingness to implement large-scale mass vaccinations or to introduce OCVs into their routine expanded programme of immunization (EPI).

## CONCLUSION

The group of experts convened in Cairo agreed on two sets of recommendations, dealing respectively with: (a) public-health use of OCVs in endemic settings, and (b) public-health use of OCVs in complex emergencies. Although several aspects of the disease itself, and of the vaccines to fight it, still need to be clarified—a concerted threshold of cholera endemicity being on the top of the list—the recommendations summarized below will guide those who consider a large-scale use of OCVs (the full text can be found in Annexure 1). Future progress in research, particularly in vaccine formulation, will call for further consultation and possible amendment of these recommendations; it will be examined in due course.

### Summarized recommendations for the use of OCVs in complex emergency settings

The relevance of oral cholera vaccination should be examined in the light of other public-health priorities. It should be linked to improved surveillance and enhanced water and sanitation programmes. A high-level commitment by all stakeholders and national authorities is critical, and a multidisciplinary approach is essential. The current internationally-available pre-qualified vaccine is not recommended once an outbreak of cholera has started and should not be performed if basic favourable conditions are not present. A decision-making tool, developed by an ad-hoc group, is intended to assist those in charge of risk assessments and subsequent decisions. This tool still needs to be validated in field conditions.

### Summarized recommendations for the use of OCVs in endemic settings

Despite the limitations of the currently-available vaccine, the use of OCVs in certain endemic situations should be recommended, and guidelines should be developed. Such use must be complementary to existing strategies for cholera control, such as safe water and sanitation, case management, and health education of the community.

Without jeopardizing the issue of recommendations, a number of topics still need to be addressed: vaccine formulation, protection against O139 serotype, and efficacy in children and in HIV-positive individuals. Surveillance data should be available to determine the best timing for vaccination, define the population at risk, and monitor the impact of interventions. Sustainable and cost-effective vaccination strategies should be decided according to each specific situation.

### Decision-making tool for use of OCVs in complex emergencies

In accordance with these recommendations, an ad-hoc meeting was organized to finalize the decision-making tool for the use of OCVs in complex emergencies that was drafted during the conference (the full text can be found in Annexure 2). A three-step approach was adopted, the relevance of OCV-use being examined at each step:

- a risk assessment for an outbreak of cholera should be undertaken first;
- an assessment of whether key public-health priorities are or can be implemented in a timely manner, combined with an analysis of the capacity to contain a possible outbreak; and
- an assessment of the feasibility of an immunization campaign using OCVs.

This document, meant to be a convenient tool, includes many practical aspects of importance when thinking of performing a vaccination campaign. Essential elements are reminded, such as the need to reinforce surveillance systems and to conduct vaccination campaigns concomitantly with other interventions, especially improvement of environmental conditions. The Global Task Force on Cholera Control, at the WHO headquarters, will provide expertise and guidance whenever necessary. Decision-makers should not hesitate to contact the Task Force with any doubts or questions, or if envisaging the use of OCVs.

This decision-making tool is now ready for field-testing. Results and feedback will be included in an adapted version. WHO is willing to coordinate with institutions or governments interested in performing such studies. If OCVs are to be used as a public-health tool, now is the moment for decision-makers—ministries of health and governments, NGOs and institutions, donors and stakeholders—to express their views of using this vaccine in the best interest of the innumerable populations affected by cholera. It cannot be conducted without advocacy, strong involvement, and resources. Sustainability, surveillance data, and a true knowledge on the burden of disease are also indispensable to effective interventions.
